# The role that composition plays in determining how a viewer looks at landscape art

**DOI:** 10.16910/jemr.13.2.13

**Published:** 2020-12-15

**Authors:** Tanya Beelders, Luna Bergh

**Affiliations:** University of the Free State, Bloemfontein, South Africa

**Keywords:** Eye-tracking, eye movements, composition, salience, landscape painting, art creation continuum

## Abstract

Viewing artworks may be subject to the same processes as everyday scene selection in respect of gaze behaviour. However, artists may employ carefully constructed composition in their paintings to lead the eyes of viewers along a predetermined path. This paper investigates whether composition is successful through comparison of expected scanpaths (constructed using the known intention of the artist) and actual scanpaths (as captured using an eye-tracker) based on a loci and sequence similarity index. The findings suggest that composition is successful in leading the eye, although the order of fixations can vary. It could thus be concluded that composition is largely successful in terms of salient elements, but less so for guiding elements. Furthermore, using Cognitive Linguistics theories and applying it to the paintings with reference to the statistical results, the Art Creation Continuum that captures the role of composition on a spectrum is proposed.

## Introduction

Composition is the “science of combination” and can be simply defined as the arrangement of elements to create a painting (1) and thus create a focal point of compositional order (2). However, the concept of composition is much more complex (3, 4) than simple arrangement and includes aspects such as the spatial relationship of elements relating to aesthetic quality (5) and addresses principles such as balance (6), movement, colour and contrast amongst others. The notion of composition has also changed over time (see 5) and encompasses perspective and geometrical shapes and placement of bodies and even includes knowledge and sensitivity of the physical position of the original artwork (2). The premise of the current study is based on the fact that every element in a picture has the potential to attract attention (7) and the overarching observation of Yarbus (8) that “composition is the means whereby the artist to some extent may compel the viewer to perceive what is portrayed in the picture”. The placement and arrangement of the elements in the picture “can be recognised only by shifting the gaze from place to place in a specific order and time” (9). It may thus be inferred that the intention of the artist is to lead the eye of the viewer along a chosen path – the placed elements essentially becoming the salient features that attract the gaze of the viewer and even cause the viewer to focus on these elements in a certain order by using for example the compositional design element of movement, thus creating what will be referred to in this paper as the compositional line. The goal of the artist is thus to ensure that the viewer will see the elements in the order planned by the artist (10). All these assertions and the premise of the study rests on the analyses of Diederot who proposed that there is an explicit link between eye movements and composition, suggesting that the composition is an “instruction to the eye, a path which the gaze follows in a certain order” (as cited in 11). Such composition could be considered analogous to bottom-up processing (in other words, visual selection that is automatically conducted based on features that are present in the scene (12)) in the sense that salient features are focused on, the difference here being that the order of fixation and salient elements are preplanned by the artist (as opposed to voluntary selection performed under control of the viewer, which is referred to as top-down selection (12)). For example, a flowing river may create the illusion of movement, causing the eye to follow the flow of the river towards the next salient feature of the painting.

Apart from the use of elements to guide the eye, other elements can be placed strategically to cause the viewer to fixate on those elements; the guiding elements then lead the eye in order to achieve the order of fixations as desired by the artist.

Therefore, composition could be an influencing factor for visual selection during the entire observation period or at the very least during the initial sweep of the painting while the viewer becomes acquainted with the elements and the scene as a whole. The premise of this study is to determine whether composition may be the overriding factor in the initial viewing behaviour of the artwork, whereafter it is possible that top-down and bottom-up processes of visual selection are employed. Regardless of whether there is a task at hand or not, if the composition is successful in leading the eye, the initial viewing of the painting should be sufficient to guide the eye along the compositional line.

## Background

As far back as 1935, Buswell (13) discovered that gaze patterns between trained and untrained viewers of art did not differ significantly but that individuals tended to fixate on the same spatial locations but not necessarily in the same temporal order. Similar to the seminal work of Yarbus (8), later confirmed by DeAngelus and Pelz (14), he discovered that the task of the viewer changes the gaze pattern significantly. Furthermore Molnar (15, 16) suggests that gaze patterns are governed by whether the viewer is looking at the picture for pleasure or to seek knowledge.

When merely looking at the painting, whether it be in a museum, book or computer, this is considered to be looking for pleasure and devoid of any task that may influence the viewing pattern – in this instance it may be the composition that is the compelling influential factor.

A number of studies have focused on this and found that the compositional design had an influence on the gaze patterns of the trained viewers – suggesting in the words of the authors that “beauty is less in the eye and more in the mind of the beholder” (17). Eye movements of viewers also differ based on preference and balance, that is, gaze patterns of viewers who preferred a balanced composition in paintings differed from those who preferred an altered or unbalanced composition. When a viewer found a painting more attractive than an alternative, where attractiveness is expressed as a sense of balance in the composition, they exhibited shorter fixations and accuracy of painting evaluation correlated with fixation duration and dwell time ( 9). However, experts and naïve art viewers can tend to focus on different elements in the painting (18).

An informal study by Gurney (19) presents some preliminary findings on eye-tracking as it relates to composition. Among these was the conclusion that placing an element in the so-called golden section - an aesthetically pleasing proportioning of the artwork as calculated by the golden ratio (20) - does not guarantee that it will draw the attention of the viewer. However, an attention-getting element, such as a face, will attract attention regardless of its position. Specifically, when painting subjects engaged in social interaction, faces attract attention while individual actions generated attention on individual body parts ( 21). This is also found when adolescents look at paintings as they display high visual attention on the human body, and give priority to elements in a painting that evoke movement or action (22). The finding that bodies or faces attract attention is in accordance with previous studies that found that viewers can spend as much as 40% of viewing time on the eye region when viewing facial photographs (23) and that faces do indeed attract attention (24). Therefore, this phenomenon is not exclusive to paintings nor, as concluded, is it as a consequence of placement, and is attributed to the fact that a person will always attempt to find a body or a face in an image and that eyes are a prominent feature of a face (25, 26). Using the same reasoning, it can be concluded that other attention-getting elements will attract attention regardless of placement and simply because a human seeks such elements in an image or scene. A second observation that was made is that no two people follow the exact same scanpath in a painting and that while the composition does not control the scanpath, it does exert an influence over the scanpath (19). Rosenberg and Klein (11) confirmed these findings, suggesting that eyes do not follow the compositional line nor do viewers scan paintings in any controlled way – that is, neither from top to bottom or left to right. There do, however, tend to be areas of interest in paintings that attract the attention of most viewers at some point (11). Most recently, Sancarlo et al., (27) found that the gaze does follow the compositional line, basing their findings on cumulative saccades instead of the gaze path, which is a different means of analysis to previous studies. Therefore, the current study can serve to formalise these findings or perhaps to discount the preliminary conclusions drawn.

In a previous study (28), eye movements within a short and prolonged period were examined. Results indicated that over a brief period, specifically 1.5 seconds, there was high similarity between how participants looked at images - but that for a longer period (3 seconds), the similarity decreases. Thus, spatial features, and perhaps the composition, of the image guide the eye during short presentations of the image.

In a recent study similar to the current study, paintings where the compositional line was known were used to determine whether the gaze indeed followed these lines. Results indicated that focal points were successful in attracting and keeping attention, but the intended entry and exit points were not used. Furthermore, the compositional line was not closely followed and the compositional elements did not exert a significant influence over eye movements (29).

Interestingly, children rely on bottom-up processing when free-viewing a painting, but revert to top-down processing when they view the painting again after being given background information. In contrast, adults rely on top-down processing both during free viewing and after being given background information on the painting (30). Under conditions where a task has been given, specifically that participants must rate the painting or give a description thereof, indicated that an initial global (bottom-up) exploration is followed by a more focused (top-down) exploration (31). These findings support the premise of this study in that, regardless of the task at hand, a successful compositional line should dominate eye movements during initial viewing.

Other studies on eye-tracking and art that do not explicitly look at composition have also been conducted. For example, gaze patterns differ based on the physical properties of the painting and whether they are viewed in realistic circumstances, such as in a museum, or on-screen ( 32). Furthermore, the number of fixations and scanpath length increase while fixation duration decreases as the painting’s level of abstraction increases (18). Both higher-level and lower-level tasks result in viewers first scanning a whole painting, followed by smaller local fixations (33). First fixation duration, total viewing time and number of fixations are all more when viewing a restored painting than the same unrestored painting (34).

## Conceptual-theoretical framework

In this article, the analysis of the eye-tracking experiment fits into the overall framework of Cognitive Linguistics as presented in Bergh and Beelders (26) and is similarly enriched by the notion of Active Vision.

Cognitive Linguistics is a branch of Linguistics and Cognitive Science that aims to account for language in accordance with the latest knowledge about the human mind, while cultural and contextual differences are also taken into consideration (35).

Cognitive Linguistics is ‘cognitive’ in the sense that, “insofar as possible, language is characterised in terms of other, more fundamental phenomena” such as memory, perception, attention and imagery (36).

The advantages of the notion of Active Vision (37) for this analysis are that it integrates seeing and looking, takes the role of eye movements into account and gives prominence to visual attention as a cognitive occurrence in “understanding perception as a dynamic process” ( 38).

The analysis in this article is based on the said model in Bergh and Beelders (26) together with further elaboration concerning it (39, 40, 41, 42) and as proposed by Gärdenfors (43), and Croft and Cruse (44). As was pointed out in Bergh and Beelders (26), conventional metaphors are usually automatic, unconscious mappings in ordinary language (“often as a result of visual perception”) and can be extended creatively (45). In Cognitive Linguistics, linguistic constructions such as words or sentences are complex cognitive models with two dimensions characterising the parameters of form and meaning (46). Given this association, they are symbolic structures that should furthermore be regarded on a continuum with other, non-linguistic constructions (47). As emphasised in Bergh (40), all manifestations and aspects of language are central to Cognitive Science research. Conceptual metaphors can also be expressed non-verbally or can motivate behaviour in terms of, say, decision-making (45, 48, 49) .

The latter point concerns especially the artists Sartore, Osner and Van der Merwe discussed below in relation to reference points in their creative way of working, which then requires them and their work to be seen in the context of research on branding (50, 51). Both the relationship with reference points and that with branding ties in with their artistic creativity in a space-time context (52), which is the main focus of Cognitive Linguistics, here then as posited in Bergh and Beelders (26).

Within the approach in Bergh and Beelders (26), the most basic, overarching metaphors have “image schemas” such as verticality and centre-periphery as their input or source domain (53).

Besides image-schematic representation, elementary structural relations also include a “profile” and a “trajector-landmark” structure ( 54), which in turn, link to attentional tuning and pop-out phenomena ( 38) and human construal, which include prominence (55). “Profiling”, that is, when a substructure is chosen for attentional focus in an expression, represents one kind of prominence (56). Another kind of prominence is known as the “salience of relational participants” – where the main figure in a scene is the relational trajector and a salient, supporting figure, the landmark, brings about the trajector (47). Related to this is Langacker’s (53) “reference-point phenomenon”, a general and basic cognitive ability that may be present in most linguistic phenomena. The way in which reference points, mental spaces and conceptual blending are related and reveals motion in the relevant model is explained in Bergh and Beelders (26). Essentially, a notion must first be activated as a suitable reference point (26), which then facilitates movement along a reference point path. Cognitive salience or characterisation of another entity serves as qualification of a notion as a reference point and the human body is an apt example of a reference point in this regard (53).

*Composition* within Bergh and Beelders (26) relates to multimodality; is included in conceptual blending; and for linguistic units is based on Langacker’s (47, 57) Cognitive Grammar views. Langacker (57) considers composition as “focussing that is inherent in the meanings of individual expressions”, which are mostly “symbolically complex, being assembled out of smaller symbolic components to form composite symbolic structures”. For example, *art* and *–ist* are symbolic components of the composite expression *artist*.

Composite expressions exhibit varying degrees of analysability; that is, they vary in how salient the component structures are in relation to the composite conception, “and how strongly they contribute to its emergence” (57). The way in which an expression’s composite meaning relates to those of its components (“at successive levels of organisation”) is called its *compositional path* - which is often shown via a tree diagram in linguistics. This means that an expression’s meaning resides in its composite semantic structure together with its compositional path in a foreground/background relationship. Langacker (57) explains it as follows: “While the composite conception is primary, it is viewed against the background of the component semantic structures at all lower levels. How strongly a particular component contributes to this secondary dimension of meaning depends on its proximity to the composite structure along the compositional path as well as the expression’s degree of analysability at the various levels.”

Langacker (57) gives three reasons for defining an expression’s meaning as including its compositional path. Firstly, it is “a very real dimension of conceptual organisation”. Secondly, it aids in explaining the general observation that “no two expressions are exactly the same in meaning”. Thirdly, “by acknowledging the semantic contribution of compositional paths, we can also explain why expressions that are *semantically anomalous* – having no coherent composite structure – nonetheless seem meaningful”.

Langacker (57) uses the terms *prominence* and *salience* interchangeably, and – like other aspects of construal, as conceptual phenomena. Even at the conceptual level, though, “the objects of our mental universe have no inherent status as profile, trajector, or landmark. These pertain specifically to the conceptualizations evoked as the meanings of linguistic expressions. How prominent a particular entity is – whether it functions as profile, trajector, landmark, or none of the above - depends on the construal imposed by the linguistic elements employed, in accordance with their conventional semantic values. … each structure in a symbolic assembly makes its own assignment of focus, so an entity focused in one structure need not have comparable salience in another” (57).

Croft and Cruse (44) provide a useful overview of linguistic construal operations as instances of general cognitive processes. The main categories are: Attention/salience; Judgement/comparison (including metaphor); Perspective/situatedness (including vantage point, orientation and deixis; and Constitution/Gestalt (including topological/geometric schematization and scale). Two aspects are of specific importance to this article, namely firstly that Croft and Cruse ( 44) point out that attention is a well-known basic phenomenon in cognitive linguistics. As such, it focusses on the human cognitive ability involved, but “there are also natural properties of phenomena in the perceived world that lend themselves to being attended to by human beings, and these properties are said to enhance those phenomena’s **salience** to human beings’ attention” and so brings about a salient-less salient continuum that relates to Langacker’s varying saliency in an overall composite structure, as mentioned earlier. An example in this regard may be a footpath. Secondly, Croft and Cruse’s ( 44) explanation of ‘here’ as opposed to ‘there’ in that “deictic elements display two layers of conceptualization: one relative to the situatedness of the speech act participants, and another construal that displaces the actual situatedness of the interlocutors to another time and place”.

One of the advantages of the framework in Bergh and Beelders (26) for this article regarding composition is that nested in multimodality and semiotics, it considers colour and movement to be semiotic modes. Because time and space are focus areas of Cognitive Linguistics (47), height, verticality, direction of movement and associated grammatical elements such as prepositions are key aspects analysed within this framework, but – as was pointed out earlier above, such linguistic symbolic structures should furthermore be regarded on a continuum with other, non-linguistic constructions (47). At such an interface, Ware (38 in 26) considers conceptual metaphors to provide a common semantic layer between verbal and visual language. Conceptual path metaphors would seem to be relevant for the analysis in this article – given the various types of paths at issue - yet most paths in this article do not represent conventional metaphors. Nevertheless, elements involved in motion along paths – such as the route - are generally relevant to the analysis based on proposals that involve pre-linguistic image schemas that structure general patters of human experience.

In considering motion along paths, and from a Cognitive Linguistics geometry perspective, Gärdenfors (43) highlights *endpoint focus* as a special case of profiling. Such a disposition could affect saliency - in that the destination is a goal. Gärdenfors (43) explains that endpoint focus can be applied to spatial phenomena (such as when a speaker’s inner gaze results in a fictive motion over a bridge) as well as processes (as in *The movie is over*, where the movie “is construed as an extended event that creates a path in time”).

For the purposes of this article, the focus is on salience – specifically then also in terms of how it relates to what may be potentially problematic in the analysis concerning points in an intended composition.

In Cognitive Linguistics, much in language are considered to be “a matter of degree”; such indistinct boundaries (47) can be dealt with optimally on continua. This also applies to saliency, which is considered to be relative.

## Aim

The aim of the study is to determine whether composition is indeed successful in guiding the gaze of the viewer. In order to determine this, the scanpath of viewers while observing a series of paintings must be inspected. The underlying assumption is that gaze behaviour may mimic general visual selection, but that the initial sweep may be subject to the guidance of the composition as composed by the artist. Therefore, it must be established whether the composition is firstly successful at guiding the eye, regardless of the motivation behind the visual selection. If composition is successful, it can further be investigated if and when the natural behaviour of visual selection starts taking precedence over composition. If composition is not successful, then it has been established that in the absence of a specific task, gaze behaviour is governed by factors independent of the arrangement of elements in a scene, or simply put, natural bottom-up selection. Therefore, the first stage of this study was to determine whether the composition guides gaze during casual viewing of a painting. The methodology therefore entailed showing a series of paintings to participants and asking them simply to look at the paintings but not giving them a specific task that could potentially influence their gaze behaviour.

## Methodology

### Eye-tracking

In order to determine whether the compositional line of a painting is successful in leading the eye of a viewer, it is necessary to capture the eye gaze of viewers while they look at a painting. In particular, fixations and saccades are important as these will allow a scanpath to be constructed and give an indication as to where the participant was looking. A saccade moves the eye to an object of interest while a fixation allows the object to be “seen”, hence the scanpath will show both the direction the eye travels in and objects that attract and keep attention. An eye-tracker makes it possible to track the gaze (58) and the software can be used to reconstruct the scanpath.

### Experimental design

The identification of the paintings included in the study was based on whether the composition of the artist (from a Western perspective) was known. Paintings with human figures and faces were excluded as it is well known that these attract attention. Art technique literature often describes the intention of the artist in order to convey the theory of composition to students. Therefore, a number of art instructional texts were used to identify paintings and the accompanying explanation, as given by the authors who were also the artists in this case, given in the text was used to construct the intended compositional line. In this manner, 35 paintings with known construction of composition (as explained by the artists themselves in their instructional texts) were chosen for inclusion in the study. This method of stimuli inclusion is similar to the Kirtley (29) study, where an instructional text was also used to identify paintings with a known compositional line. Therefore, a clear intended scanpath (as envisaged by the artist’s composition) could be extrapolated for each painting. Paintings were displayed as images on a remote eye-tracker that was used to capture eye gaze data. A Tobii TX300 eye-tracker, which has a sampling rate of 300 Hz (gaze position is captured 300 times per second), was used for the purposes of the study. The TX300 has a 23-inch widescreen monitor and used a resolution of 1920×1080. The paintings were resized such that they all fit the vertical size of the screen while maintaining the aspect ratio (all but two were resized to have a height of 1000 pixels). The paintings were shown to participants in a random sequence. Each painting was shown for five seconds as this was surmised to be sufficient time to determine whether the composition was successful in leading the eye of the participants. Tobii Studio® was used to generate gaze plots.

### Data analysis

For the scope of this paper, only a subset of the paintings will be analysed, specifically three landscape paintings in view of discussing viewing paths, as opposed to simply attention-getting elements – such as a face. The analysis was reduced to a smaller set of painting since analysing on all the included paintings would be far too time consuming and complex to compare all the paintings. Thus, it was decided to concentrate on only a few paintings that were deemed to meet meaningful criteria for substantive analysis. Therefore, these three paintings were chosen for a number of reasons, namely (i) based on the fact that they have a number of different elements in the scene and not only a single element as was found in some of the original stimuli, (ii) there was a very clear, concise intended compositional line that included numerous elements in the scene as explained by the instructional text, (iii) the paintings contained a variety of colours and not only shades of a specific colour and (iv) elements are well-defined and do not blend into other elements or the background.

Analysis of scanpaths can be conducted using a variety of methods, for instance, simple string comparison methods, vector-based methods that do not require AOIs but rather rely on geometric alignment and then the more complex algorithms that use Gaussian mixture models or hidden Markov models (see 59 for an in-depth discussion). The analysis method proposed by Privitera and Stark (60) was used. This was the first scanpath comparison that could compare both loci and order – both elements that are important to this study. This comparison method has been used in numerous studies (cf. 61, 62) and was deemed sufficiently robust to analyse the current research question.

**Figure 1.**
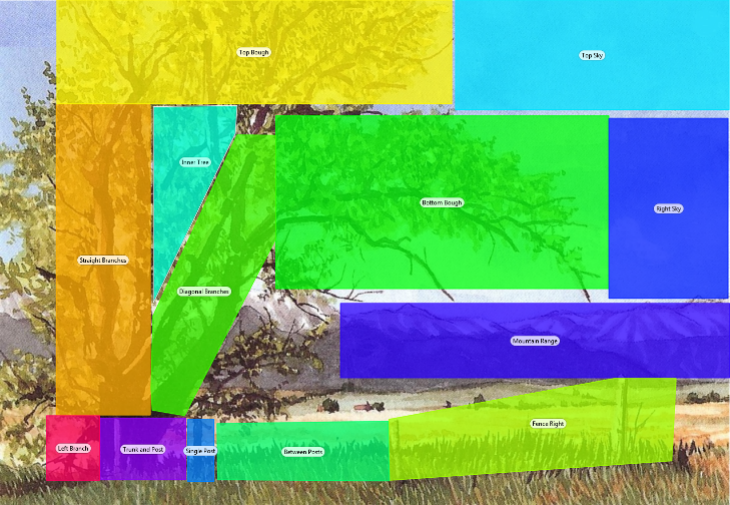
Example of compositional AOI elements

Since the chosen analysis method requires the construction of a scanpath expressed as a string, areas of interest (AOIs) were drawn on the stimuli based on the expected scanpath as potentially dictated by the composition. As previously mentioned, a scanpath could be extrapolated for each painting since the intention of the artist was explained in the literature used for identification of the included paintings – this scanpath will be referred to as the expected scanpath in this paper. Using the intended composition as explained in the instructional text, all elements that were identified as having the intention of attracting attention were designated an AOI. These AOIs were drawn on the stimuli post-test by the lead author. Figure 1 illustrates the AOIs that were superimposed over the paintings post-test. Note that the AOIs cover specific elements and are not necessarily square or uniform in size. In this example, the instructional text clearly stated the intended focal point was the tree and that the branches should guide the eye of the viewer upwards and then along the curved branches to the ground. Hence the AOIs were divided in such a manner that the upward and curved branches could be distinguished from one another as could the top branches of the tree. The area on the ground – as the final element in the compositional line – was then also subdivided into AOIs. The remainder of the AOIs were identified as potential viewing points that could have been gazed at but that were not part of the compositional line – for example the sky in the top right should the gaze of the viewer continue along the top of the tree instead of curving downwards with the branches.

Thereafter, each AOI was named using an alphabetic character, starting at A for the first expected AOI in the intended composition and proceeding alphabetically according to the order in which the AOIs were expected to be visited. For example in Figure 1, A would be the upward branch on the left of the painting, followed by the top branches as B and then the curved branches as C and so forth until the compositional line is completed as a representation of alphabetic AOIs. This allowed an expected scanpath string representation to be determined for each painting. A string representation of each individual participant’s scanpath was then constructed in the same manner, using the order in which the participant viewed the AOIs for the duration of the viewing. For example, for a specific painting there might have been 5 AOIs as identified by the composition. The expectant path would then be ABCDE. A participant might have viewed the AOIs in order CBDDEBAC. The question now is how similar the scanpath of the participant is to what the artist intended with the composition. Therefore, it is necessary to compare the expected scanpath, namely ABCDE with the actual scanpath CBDDEBAC.

Since these two scanpaths are essentially two character strings, the minimum string distance or Levenshtein distance (63) can be used for this purpose. This distance is calculated as the minimum number of corrections that must be made in order to transform one string into another (64). The operations that can be used to transform the strings are the insertion of a character (i), the deletion of a character (d) and substituting one character for another (s). This gives an integer value that represents the cost of transforming one string into another. However, scanpaths also have a direction (which is not true for character strings), which the Levenshtein distance does not account for.

Within scanpath theory, there are two indices available in order to determine how similar scanpaths are to one another, namely *S_p_*, which measures the position similarity and *S_s_*, which measures the sequence similarity (60). *S_p_* gives an indication of how closely two strings resemble one another in terms of locus (60) by determining which characters of one string are present in the other string (65). *S_s_* compares the similarity of two strings in terms of sequence (60) by calculating the cost of manipulating one string into another (65). The cost is determined as the total number of insertions, deletions and substitutions required to transform one string, in this case the expected scanpath, into another, namely the actual scanpath. This calculation is the afore-mentioned Levenshtein distance. The cost is then normalised to the length of the longer string (65) in order to compute the *S_s_* index. Consecutive fixations on the same AOI can be collapsed into a single representation in the string – this is referred to as the dwell based version of the string.

As previously acknowledged, saliency could play a pivotal role in attracting attention that the composition is moot. However, while salient features might attract attention, composition is much more than saliency, taking into account elements such as balance and spatial relationships. Additionally, compositional elements such as movement serve to guide the eye along a path, pushing it to the next element in the compositional line – something that pure saliency will not do. However, in order to investigate the effect of salient features in the painting, a saliency algorithm was used to generate random scanpaths for each painting. These were then included in the comparison analysis to distinguish between guided and random (salient-driven) gaze patterns.

Wilcoxon Rank and Mann-Whitney tests were also used to determine if the metrics differed significantly from one another. The statistical tests were conducted for the difference between the metrics of actual-expected and actual-random as well as actual-expected and random-expected scanpaths for both fixation indices. In the case of the latter tests, the dwell-based scanpaths only have been evaluated since the random scanpaths were constructed only as dwell-based.

### Participants

A convenience sample (n = 65) was used for the study as participants were employees of a bus company in the city where the study was conducted. Participation was voluntary and informed consent was given for participation. The sample consisted of both males and females of varying ages. Since the aim is to establish the role of composition in gaze behaviour which, if successful, should guide the gaze of all viewers, no demographic classification was used for analysis.

## Results

### Painting 1 – Robin’s Hood Bay

**Figure 2.**
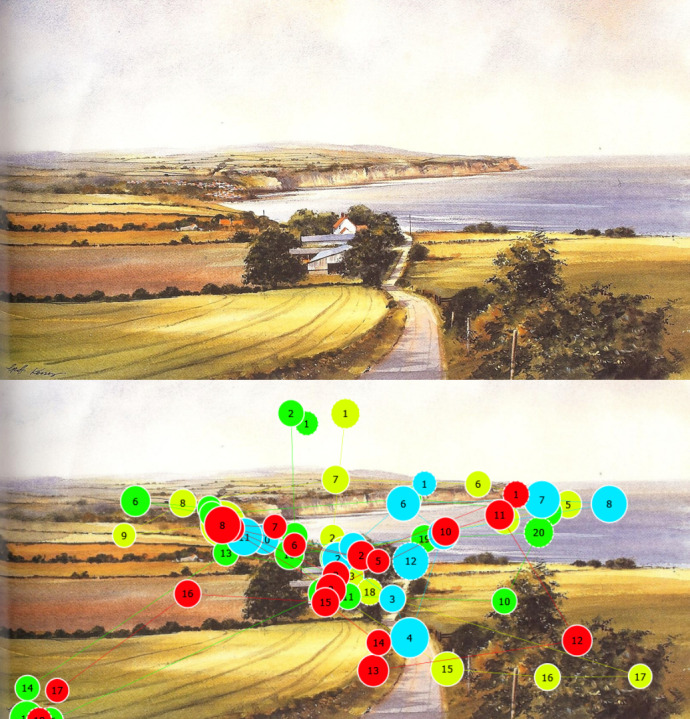
Gazeplots over Painting 1, Looking towards Robin’s Hood Bay (Source: Kersey, 2006)

The first painting analysed is titled “Looking towards Robin’s Hood Bay” and is a watercolour painted by Geoff Kersey (66). The lines of the painting (Figure 2) are subtle and were designed to make use of the road as a guiding element. The intention, as described by the artist himself, is for the eye of the viewer to enter from the bottom of the painting, move along the road as the guiding element, towards the house in the centre, where the gaze should settle (66) as the house functions as a focal point. Thereafter, it is possible that the eye will move upwards, following the coastline, and the move towards the right as it follows the cliff line out to sea.

Figure 2 shows the original painting (top) and the gaze plots of a number of participants (bottom). For the sake of clarity, only a few participants’ gaze plots are shown, but they were found to be representative of the majority of the sample. From the image it can be seen that the road, cliff, water and bushes were successful in guiding the eye. In some instances, participants glanced at the name of the artist in the bottom left corner; and in others, participants followed the line of the bushes in the bottom right corner of the painting. In order to determine whether the focal point of the house was successful in attracting and holding the attention of the viewer, a heatmap was generated. The heatmap (which is not reproduced here) clearly showed that the majority of viewing time was concentrated on the house. As per the instructional text, the house was intended as a focal point in the painting and is therefore meant to both draw and keep the attention of the viewer. This shows that the intended focal point functioned as such, although it could be attributed to the mere fact that the house is centrally located, a position known to automatically attract attention.

For comparison between actual and expected scanpaths, the mean value of *S_s_* in this instance was 0.15 (dwell-based = 0.23), while that of *S_p_* was 0.64 (dwell-based = 0.65). The low *S_s_* value combined with the higher *S_p_* indicates that the intended composition is successful in terms of standout elements, but that the order of fixations varies and does not correspond closely with the expected order.

When comparing the scanpaths of the individual participants with one another via y-matrices and parsing diagrams, the mean *S_s_* value was 0.36 (dwell-based = 0.38) and the mean *S_p_* was 0.29 (dwell-based = 0.51). Therefore, there is some similarity between the dwell-based scanpaths in terms of fixation locations and moderate similarity in terms of fixation sequence. This indicates that, to a large extent, the participants all looked at the same AOIs and sometimes in a similar order. Comparison with the randomly generated scanpaths yielded a mean *S_P_* of 0.39 (dwell-based = 0.41) and a mean *S_S_* of 0.09 (dwell-based = 0.14). The differences between the scanpaths were all significant (Table 1). Therefore, there is a significant difference between the actual-expected metrics and the random-expected metrics as well as between the actual-expected and actual-random metrics.

**Table 1:** Statistical results for countryside painting

	Actual-expected compared to actual-random	Actual-expected compared to random-expected
Dwell-based S_P_	V = 1225.0, p < 0.05	U = 97.5, p < 0.05
Dwell-based S_S_	V = 908.5, p < 0.05	U = 3672.5, p < 0.05

Based on all this evidence, it can be concluded that the intended composition was successful since most participants followed the path, albeit it in varying order of fixations - but that the elements deemed of interest were fixated upon and the guiding element leads the eye from one element to another. Additionally, the focal point was clearly successful in attracting the attention either for long periods or attracting the gaze multiple times. Therefore, the composition was shown to be successful in guiding gaze behaviour during the initial viewing of the artwork.

From a reference point stance, the house as related to human beings captured final attention, and in considering individual viewing variation, when natural *endpoint focus* (cf. 43) and a salient-less salient continuum are taken into account, the analysis of this painting can be enriched.

### Painting 2 - Trees

**Figure 3.**
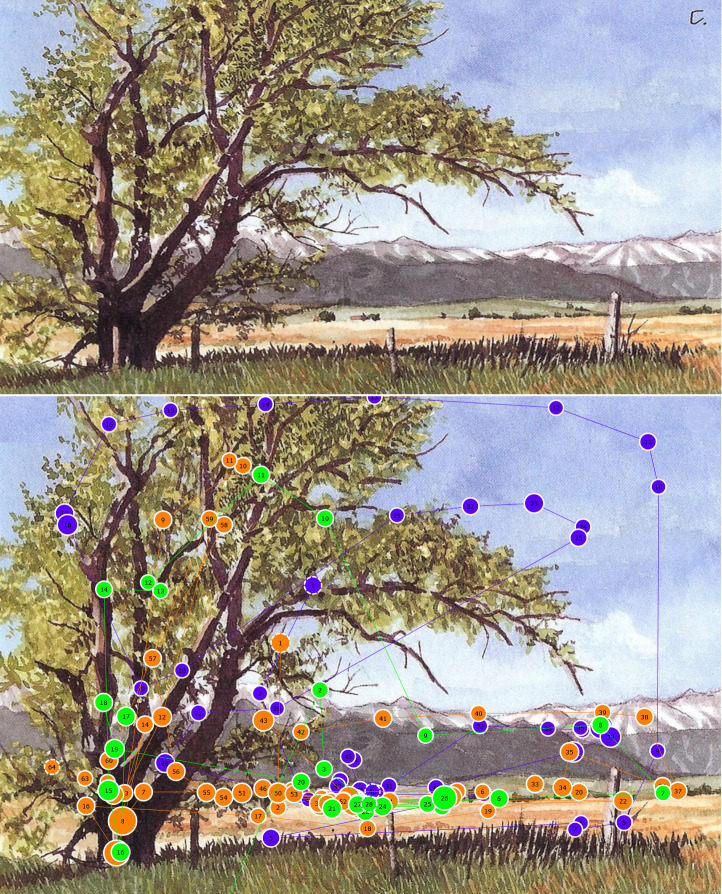
Gaze plots over Painting 2, Trees (Source: Nice, 2007)

The phenomenon of the gaze settling into a repetitive cyclic behaviour was seen on another painting (Figure 3) by Claudia Nice where the individual gaze patterns may have differed, but repetition was achieved through the placement of elements. Again, the figure shows the original painting (top) as well as a subset of gaze plots (bottom) overlaid on the painting. In this painting, the artist describes the tree as the focal point but also uses the branches to guide the viewer upwards and then along the curved branches to direct the eye towards the ground. Thereafter, the shadow of the tree is intended to guide the viewer back towards the tree – restarting the intended compositional line and causing a repetitive cyclic gaze pattern (67). The indices for this painting are similar to other paintings in this study, with a low mean *S_s_* value of 0.11 (dwell-based = 0.15) and a mean *S_p_* value of 0.48 (dwell-based = 0.50). Therefore, the same can be said; namely, that some elements attracted attention - which were not considered by the artist to form part of the intended composition, in this instance most probably the mountain range. Once again, this analysis can be complemented by taking endpoint focus and relative saliency into account.

### Painting 3 - Sheep

**Figure 4.**
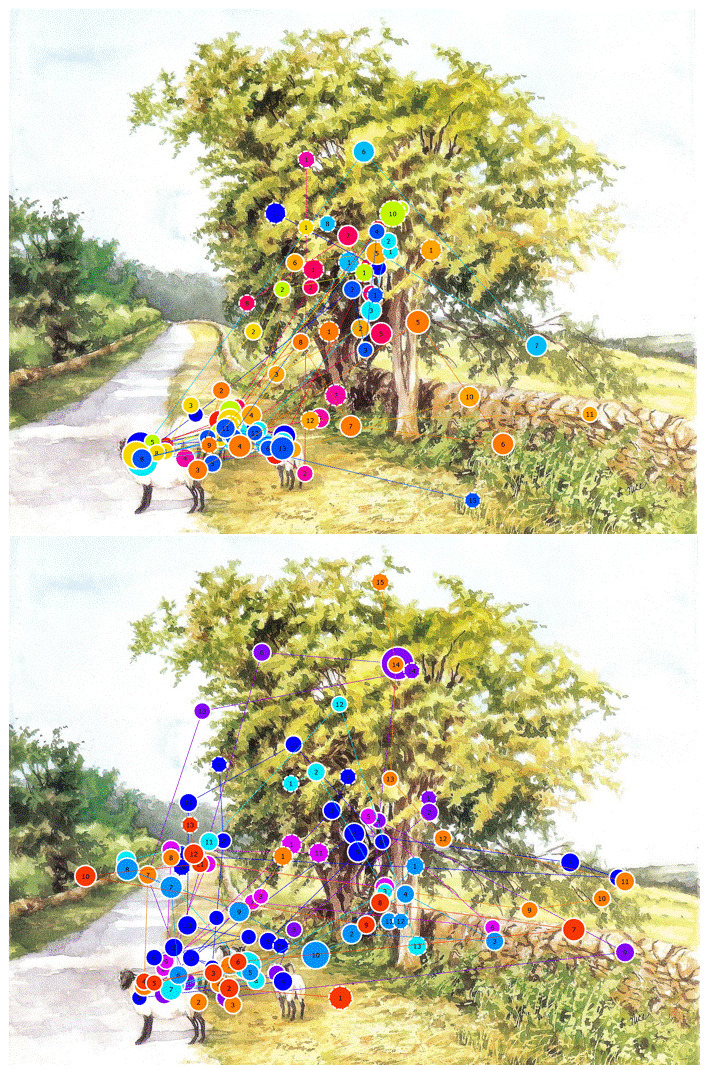
Gaze plots over Painting 3, Sheep (Source: Nice, 2007)

The sheep in Figure 4, also by Claudia Nice, below are intended to form the focal point and pull the eye towards the left after which the gaze follows the path of the country road (67). Analysis of the gaze plots showed two distinct scanpaths based on gaze behaviour of the majority of the participants. The majority of the participants followed a scanpath similar to Figure 3a, with the second tendency shown in Figure 3b. In Figure 3a, the gaze alternated between the focal points of the sheep and the trees. The path was not followed and the trees on the left were never looked at. In some instances, participants followed the line of the fence in the bottom right hand corner. Conversely, the gaze plot in Figure 3b shows that while the sheep and trees received attention as before, the path was followed as initially predicted by the compositional lines. In some instances, the trees on the left were looked at, but these are minimal. Therefore, with this painting it can be said the focal points were successful. Stated differently, endpoint focus as related to motion along a path also came into play. The mean *S_s_* value was 0.09 (dwell-based = 0.16) while the mean *S_p_* value was 0.64 (dwell-based = 0.64). These values verify the findings that the composition is successful in leading the eye, although once again the order of fixations differs. Therefore, the similarity indices show that the intended AOIs were in fact visited to a large degree by the majority of the participants.

Similarity of scanpaths were then evaluated by comparing participant’s scanpaths with one another. The mean *S_s_* index in this instance was 0.32 (dwell-based = 0.33), while the mean *S_p_* index was 0.30 (dwell-based = 0.59). Once again, the dwell-based scanpaths indicated high similarity, considerably more than the full scanpath. This is indicative of a high incidence of successive fixations within a single AOI before the eye is drawn to another AOI within the painting. The sequential similarity indicates some degree of similarity between the sequences in which AOIs were viewed.

Average indices for comparison with the randomly generated scanpath show a low sequencing similarity of 0.09 (dwell-based = 0.11) and a moderate loci similarity of 0.32 (dwell-based = 0.34). The difference between *S_S_* values of actual-expected and actual-random was significant when evaluating dwell-based scanpaths (V = 761.0, p < 0.05) as was the *S_p_* index (V = 1768.0, p < 0.05).

Comparison between actual-expected and random-expected of the dwell-based *S_S_* (U = 1696.0, p > 0.05) and *S_P_* (U = 2624.0, p < 0.05) indicated that there was a significant difference in terms of positioning but not sequencing.

## Discussion

In general, it could be said that the loci similarity was higher than the sequencing similarity, which indicated that participants looked at the same AOIs, which is to be expected, but in a different order. This confirms the oringinal findings of Buswell (13) who found spatial fixation order was similar but differed in temporal order. Thus, the path constructed by the artist is not followed in the order intended by the artist. This confirms the finding of previous studies that showed that scanpaths vary according to the viewer (68,69). These studies did, however, not account for composition. The informal study on composition indicated that placement of an element in the golden section does not guarantee that a viewer will look at the element. Since the current study did not take the golden section into account, it is difficult to determine whether this finding was confirmed or not. It can, however, be confirmed that similar to Gurney (2009), the intention of the artist as constructed by the composition (the golden section being an element of composition), plays a part in the scanpath of the viewer, but is not necessarily the overriding factor.

Since the sequencing similarity is low, but the loci similarity high, it would be beneficial to determine if any part of the expected sequence was present in the actual scanpaths. This would allow for determination on whether the guiding elements are successful, either in reverse order or dependent on where the person started looking at the painting. Future analysis could address this question that arises from the findings.

The same level of correlation was seen with comparison with the randomly generated scanpaths. In terms of loci similarity, all differed significantly from the random model for both the full scanpaths and the dwell-based scanpaths. This indicates a significant difference in terms of the AOIs that were viewed by the participants and those predicted by the model based on their saliency. The main reason for this could be attributed to the fact that more AOIs were identified and visited than were predicted by the saliency model, thus leading to a significant difference between the metrics. Furthermore, dwell-based scanpaths often had a higher similarity index than the full scanpath, thus indicating that there was a high incidence of successive fixations within a single AOI. This does not negatively impact on the success of the composition as the AOI holds the attention of the viewer as desired by the artist. In some instances, the surface area of the AOI could play a role as multiple fixations may be required to fixate on standout elements within a single AOI, such as with the branch of the tree. For each of the paintings, there was a significant difference between the actual-expected and random-expected indices, indicating that there was a significant difference between these paths. This could be indicative of the saliency model simply evaluating the prominence of elements, whereas the expected scanpath is constructed using prominent elements in the order that the intended guiding elements dictate. Similarly, this could explain the significant differences between the actual-expected and actual-random indices. Once again, the importance of integrating the salient - less salient continuum into our analysis.

Focal points succeeded in drawing the initial attention of the viewer and were, moreover, successful in drawing the gaze a number of times. Similarity indices verified that the composition was successful in terms of positional similarity, but that the ordering of fixations differed greatly. This finding can be validated in terms of our proposals regarding reference points, but moreover and also at a different level concerning the way reference points fits into the overall model of complex metaphors (26) that can also guide behaviour and decisions reached by artists about their creative process.

Focus and ordering can thus vary for viewers as well as artists.

For viewers, the first reference point on a path can be the endpoint or destination, or a picnic spot en route. Alternatively, because the journey may look dangerous, the viewer may decide not to take a particular path.

In turn, for the photographer Sartore (70), for example, the photograph is the first reference point in that a picture of a scene is taken at the starting point (70). In contrast, South African landscape artist Strijdom Van der Merwe (in 71) considers the photograph of a scene to be the final, documenting reference point.

South African art photographer Martin Osner (72) does not distinguish between art and photography, yet points out that you are not required to be an artist to be able to take an effective picture with a camera:

“The camera does not record what the eye sees – in contrast to what we expect. Focus, depth contrast, colour and lens compression change how we see the world.

“A photograph is a recording of the moment. Photography is a mechanical, technical instrument in the hands of a creative artist. But, the system is not perfect, and it is in this that lie the creative art process. If the camera is purely used in the way that it is been designed [as a perfect system], things can get quite boring. This is what makes photography so interesting. The camera is another tool on the work bench, used variously in the creative process. Knowing what to do and applying it [is the art]. The camera is just a modern inclusion in the history of art. Photography is not perfect; yet it is in this context of inconsistency that art can be realized.”

As was pointed out earlier in the analysis of Painting 1, viewers did search for the artist’s name, therefore branding does play a role in viewing a work of art. In proposing the Art Creation Continuum below in this regard, our thinking is founded in the reasoning above together with the following points made by Strijdom van der Merwe (in 71):

- Moments – Art is not required to last – what happens in a few present moments can be documented with a photograph. The experience is more important than the work of art.
- Art creation – The landscape is not imitated; material from the landscape is simply selected, used and arranged in the work of art. Personal, intimate experience with the landscape is captured in art creation. Often far away from other people, in nature. An artist is born to bring that which is deeply innate to the fore.
- Trust – with nature must be built. Landscape art creation is unpredictable in terms of time (of day), space, creation, productivity and complications such as wind and ebb and flow. It is necessary to clear the mind to capture what is seen and to create a work of art that fits the moment experienced in relation to nature as focus; harmony, not ego. Everything needed is in nature – colour, texture, designs.
- Documentation – of the work of art as a photograph to bring it to viewers, audiences and potential buyers.

Based on these points and after the analogy of our 2013 continuum in the context of visual art (cf. 51):

**Figure 5.**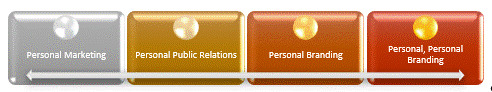-

and in keeping with the model in Bergh and Beelders (26), and following the verbal preamble (73), we propose the following Art Creation Continuum:

**Figure 6.**
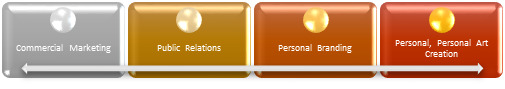
Art Creation Continuum

This continuum sees personal art creation together with art in the competitive business sector on a spectrum instead of as a binary division. Established composition may play a role in especially designing and conveying clear messages and lines when vital, whereas other forms of art may capture personal, private or sacred meaning. In building trust with nature currently, careful consideration and renewed respect in the face of climate change is essential in this treasured process.

Essentially, the continuum reveals indistinct boundaries among the categories personal-personal art creation, personal branding, public relations and commercial marketing. Predictability, consistency and chaos-order fill the in-between spaces, as the process need not always complete the full course.

Personal-personal art creation is most private and sacred in selecting compositional components and elements. This means that visibility increases from this category leftwards on the spectrum.

Three general observations about paths (November 2, 2020 Ekerk Good News e-newsletter posting by Stephan Joubert; unreferenced) illuminate the positions of artists as well as viewers on the Art Creation Continuum; namely, that no road simply dwindles somewhere; all roads also originate somewhere; and at a given point, a person joins an existing road. As in Bergh and Beelders (41), the continuum enables the visualisation of movement between the points on the spectrum. The established artists and photographers have revealed their preferred creative journey and are differentiated together with their genres in terms of the direction of their travelling, although they are bound by interfaces such as art, landscapes, photographic documentation and simplicity in their approaches. Osner (October 22, 2020 blog posting by Martin Osner to Art Gallery e-newsletter; unreferenced), for instance, reasons that “simplicity is most often key … and will always stand the test of time, and can be achieved” in various genres. “… when given a very busy composition to look at, instead of taking our time and working through each focus point of information, most of us would choose to ignore the whole photograph and just move on. I think this again is because we are continually bombarded by so much visual information on a second to second basis that we very often choose not to look at everything, again just so that we can cope”. The viewing paths of observers that join the continuum path at some point may be unknown (until revealed by eye-tracking, for example) or also confidential in being personal, personal art (re)creation, but do vary in direction – as shown by our results.

The viewers’ connection with the artist is especially through personal branding. From our stance, the viewers’ search for the artist’s signature is more than the link between text and pictures or an indication of the salience of written language, but a manifestation of a language user’s knowledge of conventions in a usage-based context endorsed by Cognitive Linguistics, as exemplified in Bergh (39). In the current environment, potential art and art photography buyers are recommended to invest in a reputable artist’s work, because quality, consistency and experience count, and to do research on the artist’s reputation, “as there is no place to hide in today’s digital society with social media and online reviews” (October 29, 2020 blog posting by Martin Osner to Art Gallery e-newsletter; unreferenced).

That much in language is a matter of degree on a continuum, “is perhaps unfortunate from the analytical standpoint – discrete entities are easier to manipulate, require simpler descriptive tools, lend themselves to stronger claims, and yield aesthetically more pleasing analyses” (47). The continuum does, however, capture the actual complexity of the data and analysis – and conclusions such as that of Sartore (70) that, “Composition (looking and thinking and deciding what you want to say with your picture and how to provide order to chaos) is the hardest part of photography”.

In the context of art creation, the continuum as confirmation of the claim that, “Because something can be salient in many different ways, describing it as such is not an adequate characterisation but only a starting point for analysis” (57). This confirmation corroborates and enforces the proposals in Bergh and Beelders (26) in terms of which salience serves as a starting point of a reference point path in a viewing path, but in this article especially as a starting point of a reference point path where an image schema (such as motion along a path) combines with a conceptual archetype (such as the use of an instrument, part-whole relationships and the natural properties of phenomena) to form complex metaphors such as Art creation is a journey.

This complex metaphor captures the conclusion that for this study and in terms of our Art Creation Continuum, a mega metaphor after the analogy of Bergh (74) and to some extent Rossouw (49) captures the overall paths to composition in the paintings concerned. As was pointed out earlier, several of the paths in our exposition are not profiled in a figurative sense, but rather technical, cognitive scientific or natural paths.

Langacker’s view mentioned above that something can be salient in many ways and that salience should therefore be merely the starting point of the analysis also corroborates our findings presented in this article on the role of composition in viewing landscape art. The Cognitive Linguistic view of relative salience is relevant in analysing especially the viewing patterns of Painting 3, but at a different level, seeing salience merely as the starting point in the analysis of the compositional line or path may reveal a further interface between visual and verbal language use - in terms of how the composite conception is viewed against the background of semantic structure at all lower levels. For example, Osner (October 22, 2020 blog posting by Martin Osner to Art Gallerye-newsletter; unreferenced) explains that Landscapes can “evoke emotion and stimulate memories of places once visited” … and that “most great compositions are achieved though simplicity. It has as much to do with what we choose to leave out … than what we decide to include. By removing elements in a composition, we create mystery and interest. Success is achieved by introducing the story and letting the viewer complete it using their imagination”. Such aspects related to “fundamental phenomena” (36) may then enhance the conceptual metaphor semantic layer that Ware (38) identifies between visual and verbal language. From a different angle, a Cognitive Linguistics analysis may then endorse the way in which variable top-down processing of guiding elements follow the initial general identification of salient elements in the compositional line in an eye-tracking study of landscape paintings.

## Limitations

Paintings were chosen based on whether the compositional path was known, a necessity for the study, but a possible limitation is that the majority of the paintings were not “busy” in the sense that there were limited elements that could draw the attention – despite the benefits of simplicity mentioned above. While this is not a detracting aspect of the study, since there was clear delineated compositional line, it would be interesting to investigate the compositional line in paintings with more potential attentional elements.

A starting gaze point was not given, rather allowing participants to have complete free viewing. Requiring a starting view point will however strengthen the results of the attractiveness of the entry points at the start of the viewing period.

The current study used only free viewing of the paintings and did not provide background information for a follow-up viewing as with Walker et al. (30). The supposition is that following the compositional line is analogous to bottom-up processing but allowing an additional viewing of the painting with added knowledge will shed more light on whether viewing patterns change accordingly.

## Conclusion

The aim of the study was to determine whether composition does in fact lead the eye of a viewer along the intended path. Viewing artwork is similar to scene selection and could therefore be subject to the same visual behaviour in terms of top-down and bottom-up processes. However, the supposition was that, due to the intended composition of the artwork, it is plausible to assume that the initial sweep of the artwork could be governed by the guidance of this composition. Thereafter, the usual gaze behaviour may take precedence. For the purposes of this paper, it was first investigated whether the composition does in fact guide the eye in any way when no specific task is given to the viewer.

The results indicate that the composition is effective in leading the eye to a large degree, generally averaging higher than 50% similarity in terms of location. This result was seen for both comparison between actual- and expected-scanpaths and between participants, more often in the dwell-based scanpaths. However, the eye did not necessarily follow in the order or direction specified. Therefore, the standout elements are successful, but the guiding elements less so. Further analysis could reveal whether the order of fixation is reversed or is present in some way to determine more definitely whether the guiding elements are successful. The eye may start at an AOI not originally intended as the first AOI, but thereafter the gaze may settle into the intended path from that point. Therefore, the intended sequence may be present somewhere within the scanpath or be reversed in some way. Analysis of this kind will also give more clarity on whether it is indeed the composition, guiding elements included, which govern gaze behaviour or whether the observed phenomenon of focusing on the standout elements is simply natural bottom-up selection. If the guiding elements are successful, then the intended sequence would appear in some form in the actual scanpath. Conversely, if it is purely natural bottom-up selection, then there may be large differences in the order in which the AOIs are visited. Comparison with a random scanpath indicated the same tendencies, namely low sequential similarity but high loci similarity indicating that the predicted AOIs were similar to the visited AOIs but, as in the case of the expected path, the order of visits to the AOI differed.

It could therefore be concluded that the composition is mostly successful in leading the eye of the viewer around the elements of the painting, albeit in varying order or direction. This strengthens prior findings (19) that composition does influence the scanpath, but does not dictate the scanpath of the viewer. The results are similar to those of Kirtley (29), confirming that focal points attract and keep attention but that the entry and exit points are largely not used as intended. Furthermore, it confirms that the compositional line guided the eye but did not dictate either order or direction. If successful, the composition could result in the eyes repeating the scanpath as constructed by the artist.

The next stage of the study would be to determine whether the guiding elements are successful, followed by an analysis on whether tasks influence this initial gaze behaviour in any way or whether top-down processes only appear once the viewer has become familiar with the composition and layout of the artwork. Factors such as prior knowledge and preference were not evaluated and could perhaps be included in further studies in order to determine whether there is interaction between these factors and the composition of the painting. Furthermore, it could be beneficial to analyse multiple viewings of the same painting in order to determine whether the observed patterns repeat with subsequent viewings.

The overall conclusion of this article is that when the eye-tracking results and statistical analyses are complemented by insights from the Cognitive Linguistics framework, apparent inconsistencies regarding salience can be accounted for in terms of endpoint-point focus in motion along a path; a salient-non-salient continuum; and immediate as opposed to distant deixis. An overarching Cognitive Science analysis incorporating Cognitive Psychology, Cognitive Linguistics and Mathematical calculations would thus be optimal. So far, our combination of these fields lead to our Art Creation Continuum, which incorporates the value of known composition lines – but also unpredictability in space and time; mood; the current increasing valued senses of touch, smell, taste and sound in aesthetic appreciation and experience (74); embodied human emotion and memories (74); and viewing variation – all essential for making and seeing art.

## Ethics and Conflict of Interest

The author(s) declare(s) that the contents of the article are in agreement with the ethics described in http://biblio.unibe.ch/portale/elibrary/BOP/jemr/ethics.html and that there is no conflict of interest regarding the publication of this paper. The data was collected by the researchers before their university had a formal ethics clearance committee but it was collected according to strict ethical guidelines nonetheless.
